# A fluorogenic substrate for the detection of lipid amidases in intact cells

**DOI:** 10.1016/j.jlr.2024.100520

**Published:** 2024-02-17

**Authors:** Mireia Casasampere, Johnson Ung, Alejandro Iñáñez, Carine Dufau, Kazuhito Tsuboi, Josefina Casas, Su-Fern Tan, David J. Feith, Nathalie Andrieu-Abadie, Bruno Segui, Thomas P. Loughran, José Luis Abad, Gemma Fabrias

**Affiliations:** 1 Department of Biological Chemistry, Research Unit on BioActive Molecules, Institute for Advanced Chemistry of Catalonia (IQAC-CSIC), Barcelona, Spain; 2 Division of Hematology and Oncology, Department of Medicine, University of Virginia School of Medicine, Charlottesville, VA, USA; 3 Department of Microbiology, Immunology and Cancer Biology, University of Virginia School of Medicine, Charlottesville, VA, USA; 4 INSERM UMR 1037, Cancer Research Center of Toulouse (CRCT), Toulouse, France; 5 Equipe Labellisée Fondation ARC pour la recherche sur le cancer, Toulouse, France; 6 Department of Pharmacology, Kawasaki Medical School, Kurashiki, Okayama, Japan; 7 CIBEREHD, Madrid, Spain; 8 University of Virginia Cancer Center, University of Virginia School of Medicine, Charlottesville, VA, USA; 9 Université Toulouse III - Paul Sabatier, Toulouse, France; 10 Spanish National Research Council (CSIC)’s Cancer Hub, Madrid, Spain

**Keywords:** sphingolipids, ceramides, lipids, enzymology, chemical synthesis, *N*-palmitoylethanolamine, anandamide, ceramidases, fatty acid amide hydrolase, *N*-acylethanolamine acid amidase

## Abstract

Lipid amidases of therapeutic relevance include acid ceramidase (AC), *N*-acylethanolamine-hydrolyzing acid amidase, and fatty acid amide hydrolase (FAAH). Although fluorogenic substrates have been developed for the three enzymes and high-throughput methods for screening have been reported, a platform for the specific detection of these enzyme activities in intact cells is lacking. In this article, we report on the coumarinic 1-deoxydihydroceramide RBM1-151, a 1-deoxy derivative and vinilog of RBM14-C12, as a novel substrate of amidases. This compound is hydrolyzed by AC (^app^*K*_m_ = 7.0 μM; ^app^*V*_max_ = 99.3 nM/min), *N*-acylethanolamine-hydrolyzing acid amidase (^app^*K*_m_ = 0.73 μM; ^app^*V*_max_ = 0.24 nM/min), and FAAH (^app^*K*_m_ = 3.6 μM; ^app^*V*_max_ = 7.6 nM/min) but not by other ceramidases. We provide proof of concept that the use of RBM1-151 in combination with reported irreversible inhibitors of AC and FAAH allows the determination in parallel of the three amidase activities in single experiments in intact cells.

Ceramidases hydrolyze the amide bond of several sphingolipids to release the corresponding amines and fatty acids. In mammals, five different ceramidases have been identified: acid ceramidase (AC), neutral ceramidase (NC), and alkaline ceramidases (ACER1-3). They are encoded by five different genes (*ASAH1*, *ASAH2*, *ACER1*, *ACER2*, and *ACER3*, respectively) and differ in the pH required for optimal activity (acid, neutral, and alkaline), substrate selectivity, and subcellular localization (1). Mutations in two of these genes, namely *ASAH1* and *ACER3*, cause rare inherited diseases. Farber disease (FD) and spinal muscular atrophy with progressive myoclonic epilepsy are both caused by mutations in the *ASAH1* gene (2), whereas *ACER3* mutations provoke progressive leukodystrophy in early childhood (3). In addition, the involvement of ceramidases in cancer progression and resistance to therapy is well documented. Several reports identified a role of AC in prostate cancer (4, 5, 6), melanoma (7, 8, 9, 10), and acute myeloid leukemia (AML) (11, 12, 13). In addition, NC regulates colon cancer initiation and development (14), ACER3 promotes growth of hepatocellular carcinoma cells (15) and supports AML survival (16), and ACER2 is involved in hepatocellular carcinoma (17, 18) and estrogen receptor-positive breast tumorigenesis (19).

Other lipid amidases of physiological relevance and importance as therapeutic targets include fatty acid amide hydrolase (FAAH) and *N*-acylethanolamine-hydrolyzing acid amidase (NAAA). Both are responsible for the hydrolysis of *N*-acylethanolamines (NAEs) to regulate their endogenous levels and effects. FAAH is a serine hydrolase and the principal enzyme for the degradation of endocannabinoids (20). The unique position of FAAH in modulating the function of diverse endocannabinoids associated with various disease conditions, including pain, inflammation, neurodegeneration, depression, anxiety, and others, has attracted significant attention to the enzyme as a therapeutic target (21). NAAA is a lysosomal hydrolase that preferentially catabolizes *N*-palmitoylethanolamine (PEA) at acidic pH (22). Several lines of evidence support that NAAA contributes to the induction and maintenance of both acute and chronic inflammation. The fact that such effects depend, in a majority of cases, on the activation of PPAR-α by endogenously produced PEA suggests that NAAA activity might promote inflammation and delay resolution by suppressing PEA-dependent PPAR-α activation (23). Furthermore, since PEA is known to have analgesic properties, NAAA inhibitors were assessed in several models of neuropathic pain (24). Importantly, the primary structure of NAAA exhibits 33–35% amino acid identity to that of AC, and both belong to the choloylglycine hydrolase family. Furthermore, Tsuboi *et al.* (25) demonstrated the ability of AC to hydrolyze NAEs and suggested a physiological role for AC as a third NAE hydrolase.

The translational interest of the above lipid amidases reinforces the need to develop specific probes for diagnostic applications and drug discovery library screening. In both instances, intact cells are preferable over cell-free systems. For diagnostics, cells isolated from liquid biopsies can be directly processed, thereby shortening the analysis time and decreasing the risk of protein degradation. In drug discovery, phenotypic screening in living cells takes into account complex biological processes absent from cell-free systems (i.e., cell uptake, metabolism, and subcellular compartmentalization and concentration of potential drug candidates).

We previously reported a method for the specific monitoring of AC activity in intact cells by flow cytometry using an azido-functionalized AC substrate (RBM5-177) together with a fluorescent reagent for click-based metabolic labeling (26). In this article, we report the functional characterization of a new AC substrate, namely RBM1-151, to monitor AC activity in intact cells by fluorimetry upon processing of the resulting product by oxidation and β-elimination. RBM1-151 is not hydrolyzed by any other ceramidase, but it is deamidated by FAAH and NAAA. Combining the use of RBM1-151 with specific irreversible inhibitors of AC (chloroacetyl(dihydro)sphingosine; SACLAC [(2*S*,3*R*)2-chloro-*N*-(1,3-dihydroxyoctadecan-2-yl)acetamide] or SOCLAC [(2*S*,3*R*,*E*)2-chloro-*N*-(1,3-dihydroxyoctadec-4-en-2-yl)acetamide]) (27) and FAAH (URB597) (28) allows for the estimation of AC, NAAA, and FAAH activities in a single experiment using cell-free systems and intact cells. We also report on the use of this probe to screen for AC activity in a panel of AML and melanoma cell lines.

## Materials and methods

### Materials

For FD, FD/AC, HEK293, HeLa T-REX, and *ASAH2*^(−/−)^ mouse embryonic fibroblasts (MEFs) and melanoma cell lines, Dulbecco's modified Eagle's medium, FBS, penicillin/streptomycin solution, nonessential amino acids, tetracycline, and puromycin were from Sigma. Zeocin was from Genaxxon Bioscience, lasticidin was from CalBiochem, and hygromicin B was from Invitrogen. For AML cell lines, RPMI-1640 medium and FBS were obtained from Corning (ref. 10-040) and VWR (ref. 97068-085), respectively. Recombinant human NC (rhNC) was obtained from R&D Systems. URB597 was from Sigma-Aldrich (ref. 341249), and ARN726 was from Tocris (ref. 5861). Primary antibodies used were AC (BD Biosciences, ref. 612302; Research Resource Identifier [RRID]: AB_399617) and β-actin (Cell Signaling Technology, ref. 3700; RRID: AB_2242334). Secondary antibody used was HRP-linked horse anti-mouse (Cell Signaling Technology, ref. 7076; RRID: AB_330924). All antibodies were prepared as per the recommended manufacturer’s protocol. SACLAC and SOCLAC were synthesized in our laboratories. Since both are irreversible AC inhibitors with similar activities (supplemental Fig. S1), they were used interchangeably in this work.

### Cells

FD, FD/AC, A375/WT, A375/AC, C8161, WM9, LNCaP, HEK293/WT, HEK293/NAAA, HeLa T-REX, and *ASAH2*^(−/−)^ MEFs were cultured at 37°C and 5% CO_2_ in Dulbecco's modified Eagle's medium high glucose supplemented with 10% FBS and 1% penicillin/streptomycin solution. LNCaP were cultured in RPMI-1640 medium supplemented with 10% FBS, 1% penicillin/streptomycin solution, 1% Hepes, and 1% sodium pyruvate. Nonessential amino acids (0.1 mM) were also added to HEK293 and LNCaP cells. The antibiotic selection of A375/AC was performed with blasticidin (3 μg/ml) and hygromycin (250 μg/ml). Ectopic expression of AC was induced with doxycycline at 1 μg/ml for 24 h before use. Zeocin (25 μg/ml) and blasticidin (5 μg/ml) were also added to HeLa T-REX cells (HeLa T-REX-human ACER [*hACER*]2, HeLa T-REX-mouse ACER [*mAcer*]1, and control vector HeLa T-Rex pcDNA4). *mAcer1* and *hACER2* gene expression was induced by adding 10 ng/ml tetracycline to the medium. Antibiotics other than penicillin/streptomycin were removed during treatments. The 501Mel cell line was maintained in RPMI-1640 medium supplemented with 10% FBS, 1% l-glutamine (200 mM), and 1% penicillin–streptomycin (10,000 units/ml). AML cells were grown in RPMI-1640 medium supplemented with 20% FBS. All cell lines were incubated at 37°C and 5% CO_2_ in a humidified incubator.

### Amidase activities in cell-free systems

All in vitro assays were conducted in 96-well plates at a final volume of 100 μl/well using reported procedures for AC (29), NAAA (30), and FAAH (31). The specific enzyme sources and reaction conditions are summarized in supplemental Table S1. Enzyme sources were AC, lysates from A375/AC (20 μg/well); NC, rhNC (5 ng/well); ACER3, lysates of ASAH2^(−/−)^ MEFs (140 μg/well); ACER1 and ACER2, microsomes of HeLa T-Rex cells stably overexpressing mACER1 and hACER2 (32) (100 μg/well); FAAH, lysates (25 μg/well) or microsomes (50 μg/well) of LNCaP cells or *ASAH2*^(−/−)^ MEFs, respectively; NAAA, lysates of HEK293 cells transiently overexpressing human NAAA (5–10 μg/well). Cell lysates and microsomes were prepared as reported (29). Reaction buffers were AC and NAAA, 25 mM acetic/acetate buffer (pH 4.5); NC, 50 mM Hepes, 150 mM NaCl, 1% sodium cholate (pH 7.4); ACERs, 50 mM Hepes, 1 mM CaCl_2_ (pH 9.0); FAAH, 50 mM Hepes, 1 mM EDTA, 0.1% BSA (pH 7.4). For the determination of *K*_m_ and *V*_max_ (AC, NAAA, and FAAH), serial dilutions of substrates in the appropriate reaction buffer were made from 200 μM solutions prepared from 4 mM stock solutions in ethanol. The reaction mixtures were incubated at 37°C for 3 h, except for the determination of *K*_m_ and *V*_max_ (30 min). In all cases, reactions were stopped with 25 μl/well of methanol and then 100 μl/well of NaIO_4_ (2.5 mg/ml in 100 mM glycine-NaOH buffer [pH 10.6]) was added. After incubation at 37°C for 1 h in the dark, 100 μl/well of 100 mM glycine-NaOH buffer (pH 10.6) was added, and fluorescence was measured spectrophotometrically at excitation and emission wavelengths of 355 and 460 nm, respectively (33). The same reaction mixtures without enzymes were used as blanks. Inhibitors were added at the indicated concentrations 15–60 min prior incubation with the substrate (10 min for HEK293 cell lysates).

### Amidase activity in intact cells

Cells were plated in 96-well plates at 20,000 cells per well with 25 μl in the appropriate medium (see above). SACLAC, SOCLAC, and DMSO were prepared in medium supplemented with 20% FBS at the appropriate concentrations from a DMSO stock solution, and 25 μl were dispensed onto the 96-well plate. Cells were incubated with the indicated concentrations of SACLAC, URB597, or DMSO (vehicle control) for 1 h or the indicated times in a humidified incubator at 37°C and 5% CO_2_. For time-course studies, cells were resuspended in inhibitor-free culture medium before RBM1-151 incubation. Unless indicated otherwise, after incubation with the inhibitors, 50 μl of RBM1-151 was dispensed onto the cells at a final concentration of 20 μM in medium with 20% FBS. Cells were incubated with RBM1-151 for 1 h in a humidified incubator at 37°C and 5% CO_2_. After RBM1-151 incubation, 25 μl of 100% methanol was added to each well. Immediately after, 100 μl of 2.5 mg/ml sodium periodate in 100 mM glycine (pH 10.6) was added to each well. The plate was incubated in a humidified incubator at 37°C and 5% CO_2_ for 30 min. Fluorescence was measured at 355 nm excitation and 460 nm emission using a microtiter plate reader. During data analysis, background signal from RBM1-151 in culture media without cells was subtracted from all values, and each amidase activity was calculated using the following equation: [umbelliferone]_C_ - [umbelliferone]_I_, where [umbelliferone] corresponds to the amounts in μM/h/2 × 10^4^ cells produced in both control cells (C, treated with DMSO) and cells treated with each inhibitor (I) calculated from an umbelliferone calibration curve.

### Western blot analysis

Cells were pelleted at 400 *g* for 10 min, washed in 1× PBS, and pelleted in a microfuge tube at 700 *g* for 7 min at 4°C. The supernatant was removed, and the pellets were resuspended in 125 μl RIPA buffer (Sigma; ref. R0278-500Ml) and incubated at 4°C for 30 min. Samples were centrifuged at 16,000 *g* at 4°C for 10 min. After incubation, the supernatant was removed and collected into labeled microfuge tubes. Protein quantification was assessed using the BCA protein assay kit (Pierce; ref. 23225) following the manufacturer’s protocol. Samples were prepared in sample buffer (Invitrogen; ref. NP0007), reducing agent (Invitrogen; ref. NP0009), and heat inactivated at 90°C for 10 min. Protein samples were resolved on a Bolt 4–12% Bis-Tris Plus SDS-PAGE gel (Invitrogen; ref. NW04122BOX) and transferred to LF-PVDF membranes (BioRad; ref. 10026934). Membranes were reactivated in 100% methanol for 1 min and blocked for 1 h at room temperature in 5% nonfat milk. Primary antibodies were incubated on a rocker overnight at 4°C. The membranes were washed three times in 1× Tris-buffered saline with 0.1% Tween-20 and incubated with the secondary antibody for 1 h at room temperature. To assess relative ASAH1 and β-actin protein levels, the membranes were incubated in SuperSignal West Femto Maximum Sensitivity Substrate (Bio-Rad; ref. 34096) and Clarity Max Western ECL Substrate (Bio-Rad; ref. 1705062), respectively and visualized by chemiluminescence using the Bio-Rad ChemiDoc MP imaging system. Protein quantification was analyzed using the Bio-Rad ImageLab 6.0.1 software.

### UPLC-HRMS

Treatments, lipid extraction, and instrumental analyses were carried out following the reported procedures (26).

### Statistics

Comparison between two means has been carried out with the unpaired two-tailed *t*-test. For comparison of more than two means, data have been analyzed by one-way ANOVA followed by Tukey multiple comparison test. To determine *K*_m_ and *V*_max_, the curves corresponding to the amount of umbelliferone produced as a function of RBM1-151 substrate concentration in the presence of different enzyme sources were determined by the Michaelis-Menten equation provided in GraphPad Prism 6 (GraphPad Software, Inc).

## Results

### Structure and synthesis of RBM1-151

**Scheme 1:**
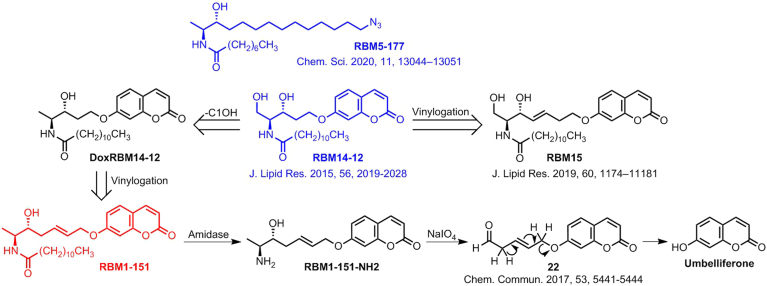
Design of RBM1-151. The original compounds that inspired RBM1-151 (RBM5-177 and RBM14-C12) are in blue. Production of umbelliferone from RBM1-151 by amide hydrolysis and further in situ oxidation and β-elimination.

In our aim to identify specific AC substrates with diagnostic interest, we uncovered that the C1-deoxydihydroceramide RBM5-177 (Scheme 1) was hydrolyzed by AC, but not by other ceramidases (26), suggesting that removal of the C1OH function provided very high AC selectivity. On the other hand, compound RBM14-C12 (Scheme 1) was reported as a fluorogenic AC sensor (34), but it was also hydrolyzed by NC and ACER3 (35). Both findings prompted us to propose that DoxRBM14-C12, the C1-deoxyderivative of RBM14-C12 (Scheme 1), might be a specific fluorogenic AC substrate. This compound was synthesized, but it was not hydrolyzed by AC, indicating that the C1OH function was required in RBM14-C12 for AC hydrolysis. Since the RBM14 vinylogs RBM15 were fluorogenic substrates of ceramidases (29), compound RBM1-151, a vinylog of DoxRBM15 (Scheme 1), was anticipated as a putatively specific AC substrate. The double bond was located at the C5 position because β-elimination of aldehyde **22** to produce umbelliferone occurs with higher efficiency under milder conditions than from its isomer at C2 (36). In this case, the retro oxa-Michael reaction required for the release of the fluorescent reporter could be promoted under basic pH conditions by abstraction of one of the α-protons of the intermediate aldehyde (36) resulting from NaIO_4_ oxidation of the aminoalcohol RBM1-151-NH2 (**8**) arising from hydrolysis of the probe by the target amidase (Scheme 1).

**Scheme 2:**
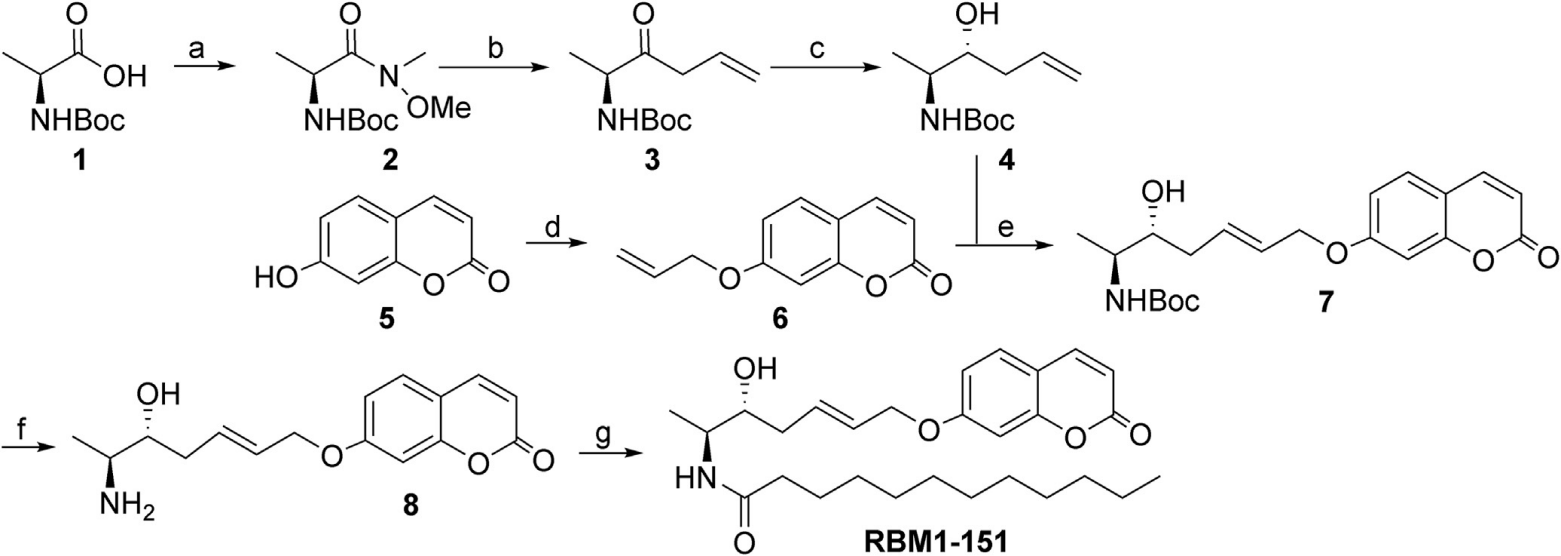
Synthesis of RBM1-151. Reagents and conditions: *a*) *N*,*O*-dimethylhydroxylamine (HCl), EDC·HCl, NMM, CH_2_Cl_2_, −15°C to room temperature, 4 h, 97%; *b*) Allylmagnesium bromide, THF, −78°C to room temperature, 3 h, 78%; *c*) LiAlH(O*t*Bu)_3_, EtOH, −78°C to 0°C, 3 h, 77% (dr = 96:4); *d*) allyl bromide, acetone, reflux; *e*) second G Grubbs catalyst, CH_2_Cl_2_, 6 h, reflux, 50%; *f*) CH_3_COCl, MeOH, 24 h, 88%; *g i*) NEt_3_, CH_2_Cl_2,_*ii*) lauric acid, HOBt, EDC·HCl, CH_2_Cl_2_, 70%.

The synthesis of RBM1-151 was carried out as shown in Scheme 2 (details given in Supplemental data). The key step was a cross metathesis reaction between the allyl derivatives **4** and **6**, which were obtained following reported procedures for **4** (37) and **6** (38). In the latter, the required (2*S*,3*R*)-*erythro* stereochemistry was achieved with excellent diastereoselectivity by reduction of **3** with LiAlH(^t^BuO)_3_. Enantiopure **7**, isolated by flash chromatography, was sequentially submitted to Boc deprotection and *N*-acylation with lauric acid to obtain RBM1-151 in high yield.

### RBM1-151 is hydrolyzed by AC, FAAH, and NAAA in cell-free systems

**Fig. 1:**
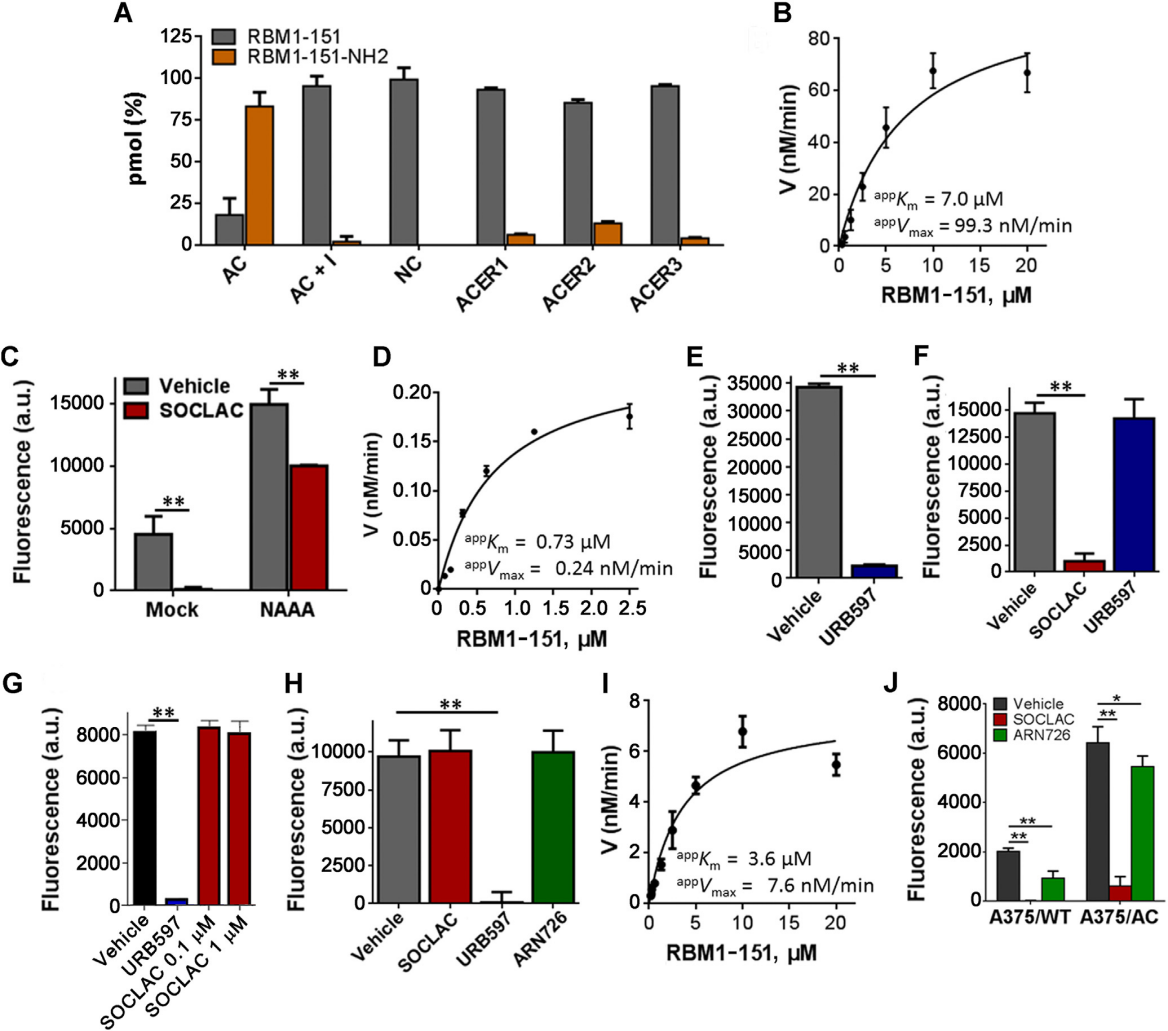
Hydrolysis of RBM1-151 in cell-free systems. A: RBM1-151 (20 μM) was incubated for 1 h in the appropriate buffer with recombinant NC (5 ng), lysates of A375/AC cells (20 μg) (AC) or *ASAH2-*null MEFs (140 μg) (ACER3) or microsomes (100 μg) from HeLa TRex ACER1 (*ACER1*) or HeLa TRex ACER2 cells (*ACER2*) induced with tetracycline, and the lipid extracts were analyzed by UPLC-HRMS. SOCLAC (I) (1 μM) was added to the AC375/AC lysate (AC + I) 1 h prior substrate addition. B: Hydrolysis of different concentrations of RBM1-151 by A375/AC cell lysates (20 μg) in acid buffer. Incubation time was 30 min. Michaelis-Menten analysis gave ^app^*K*_m_ = 7.0 μM; ^app^*V*_max_ = 99.3 nM/min. C: Lysates of HEK293/NAAA or HEK293/mock (10 μg protein) were incubated for 3 h with RBM1-151 (5 μM) at acid pH in the presence or the absence (vehicle) of SOCLAC (1 μM), and the reaction mixture was processed for fluorescence release as detailed in the Materials and methods section. D: Hydrolysis of different concentrations of RBM1-151 by SOCLAC-pretreated HEK293/NAAA cell lysates (5 μg). Incubation time was 3 h at acid pH. Michaelis-Menten analysis gave ^app^*K*_m_ = 0.73 μM; ^app^*V*_max_ = 0.24 nM/min. E: Lysates (25 μg protein) of LNCaP cells were incubated for 3 h with RBM1-151 (10 μM) in neutral buffer B in the presence or the absence (vehicle) of URB597 (50 μM), and the reaction mixture was then processed for fluorescence release as detailed in the Materials and methods section. F: Lysates (25 μg protein) of LNCaP cells were incubated for 3 h with RBM1-151 (10 μM) at acid pH in the presence or the absence (vehicle) of URB597 (50 μM) or SOCLAC (0.1 μM), and the reaction mixture was then processed for fluorescence release as detailed in the Materials and methods section. G: Microsomes (50 μg protein) from LNCaP cells were incubated for 3 h with RBM1-151 (10 μM) in neutral buffer B in the presence or the absence (vehicle) of URB597 (50 μM) or SOCLAC, and the reaction mixture was then processed for fluorescence release as detailed in the Materials and methods section. H: Lysates (25 μg protein) of *ASAH2-*null MEFs were incubated for 3 h with RBM1-151 (10 μM) in neutral buffer B in the presence or the absence (vehicle) of URB597 (50 μM), SOCLAC (0.1 μM), or ARN726 (0.1 μM), and the reaction mixture was then processed for fluorescence release as detailed in the Materials and methods section. I: Hydrolysis of different concentrations of RBM1-151 by *ASAH2*-null MEF lysates (20 μg). Incubation time was 30 min. Michaelis-Menten analysis gave ^app^*K*_m_ = 3.6 μM; ^app^*V*_max_ = 7.6 nM/min. J: Lysates of A375/WT and A375/AC cells (20 μg) were incubated for 3 h with RBM1-151 (10 μM) in acid buffer in the presence or the absence (vehicle) of SOCLAC (0.1 μM) or ARN726 (0.1 μM), and the reaction mixture was then processed for fluorescence release as detailed in the Materials and methods section. Data (mean ± SD) were obtained from two (A, C, D, and J) or three (B, E, F, G, and H) different experiments with triplicates. Statistical significance between means was analyzed by one-way ANOVA followed by Tukey multiple comparison test. Asterisks indicate statistical significance at ∗*P* ≤ 0.001 and ∗∗*P* ≤ 0.0001. The enzyme sources and buffers used for each enzyme are summarized in supplemental Table S1. That SOCLAC and SACLAC have almost identical activity as AC inhibitors is shown in supplemental Fig. S1. AC, acid ceramidase; MEF, mouse embryonic fibroblast; NAAA, N-acylethanolamine-hydrolyzing acid amidase; NC, neutral ceramidase; SACLAC, (2*S*,3*R*)2-chloro-*N*-(1,3-dihydroxyoctadecan-2-yl)acetamide; SOCLAC, (2*S*,3*R*,*E*)2-chloro-*N*-(1,3-dihydroxyoctadec-4-en-2-yl)acetamide.

To investigate whether RBM1-151 was hydrolyzed by each of the five known ceramidases, the probe was tested on lysates from human AC-overexpressing melanoma A375 cells (A375/AC), rhNC, microsomes from HeLa overexpressing tetracycline-induced ACER1 or ACER2 (26), and lysates from *ASAH2*-null MEFs as a source of ACER3 (35) using the appropriate buffers (supplemental Table S1). UPLC-HRMS analysis of lipid extracts was carried out first to confirm the identity of the reaction product (RBM1-151-NH2) (supplemental Fig. S2). These analyses revealed that RBM1-151 was only hydrolyzed by AC since the expected free amine (RBM1-151-NH2) was abundantly detected in lipids from A375/AC cells and poorly by ACER-overexpressing cells (Fig. 1A and supplemental Fig. S2). Furthermore, formation of the free amine in A375/AC cells was abrogated by preincubation with SOCLAC, an irreversible AC inhibitor (Fig. 1A) (27). Once the identity of the RBM1-151 deamidation product (RBM1-151-NH2) was unambiguously confirmed by UPLC-HRMS, the final goal was to analyze RBM1-151 hydrolysis by measuring the amount of umbelliferone generated from RBM1-151 by mild in situ oxidation of the RBM1-151NH2 reaction product. This is a faster and easier analytical technique as compared with UPLC-HRMS, which requires costly equipment not available in all laboratories. Furthermore, in contrast to the UPLC-HRMS technique, the fluorescence assay can be carried out in a high content format (96- or 384-well plates). Kinetic experiments carried out by fluorescence measurement using A375/AC cell lysates showed that RBM1-151 was hydrolyzed by AC with ^app^*K*_m_ and ^app^*V*_max_ values of 7.0 μM and 99.3 nM/min, respectively (Fig. 1B).

Given the structural and mechanistic similarities between AC and NAAA, hydrolysis of RBM1-151 was tested in lysates of NAAA-overexpressing HEK293 cells (HEK293/NAAA). As depicted in Fig. 1C, significantly higher amounts of fluorophore were produced from RBM1-151 in HEK293/NAAA than in mock cells (HEK293/mock), showing that RBM1-151 is a substrate of NAAA. Although *N*-(4-methylcoumarin)palmitamide was reported as a fluorogenic NAAA substrate (39), whether this compound was also hydrolyzed by AC and FAAH was not investigated. On the other hand, in HEK293/mock cells, the RBM1-151 hydrolytic activity was remarkably decreased by preincubation with SOCLAC, suggesting that basal activity of HEK293 cells over RBM1-151 at acidic pH is due to AC. Importantly, SOCLAC reduced umbelliferone production from RBM1-151 in both HEK293/mock and HEK293/NAAA to similar extents, which supports that SOCLAC does not inhibit NAAA. This is important in the intact cell studies, shown below, where SOCLAC and URB597 are used to discern between AC, NAAA, and FAAH activities. Using SOCLAC-pretreated HEK293/NAAA cell lysates in which NAAA is the only active lipid amidase at acidic pH, kinetic analysis of RBM1-151 hydrolysis by NAAA afforded ^app^*K*_m_ and ^app^*V*_max_ values of 0.73 μM and 0.24 nM/min, respectively (Fig. 1D).

To investigate the capacity of FAAH to hydrolyze RBM1-151, the probe was incubated with lysates from LNCaP cells, reported to express high FAAH levels (40), in FAAH buffer at neutral pH. As shown in Fig. 1E, LNCaP cell lysates produced fluorescence from RBM1-151, and this fluorescence was significantly decreased (13% of vehicle remaining) by incubation with the irreversible FAAH inhibitor URB597 (28). Although LNCaP cells contain high AC levels (41), URB597 did not reduce umbelliferone production from RBM1-151, but SOCLAC did, in LNCaP cell lysates at acid pH (Fig. 1F). Moreover, SOCLAC at 1 and 0.1 μM (0.1 μM was routinely used to save material) did not modify umbelliferone generation from RBM1-151 in LNCaP microsomes at neutral pH, whereas URB597 prevented the fluorophore release (Fig. 1G). These results demonstrate that URB597 does not inhibit AC and that SOCLAC does not inhibit FAAH. In addition, URB597 has been reported not to inhibit NAAA (42). Overall, these data confirm that RBM1-151 is a FAAH substrate and that, in combination with URB597, it can be used to measure FAAH activity. Although a fluorogenic FAAH substrate has been reported (43), whether it was also hydrolyzed by AC and NAAA was not investigated. Kinetic constants of RBM1-151 hydrolysis by FAAH were determined next. These experiments were carried out in *ASAH2*-null MEF lysates at neutral pH, in which RBM1-151 hydrolysis was blocked with URB597, but not with either SOCLAC or ARN726, a reported irreversible NAAA inhibitor (Fig. 1H). Hence, in these cells, only FAAH activity is measured at neutral pH with RBM1-151. Using this cell line, FAAH hydrolyzed RBM1-151 with ^app^*K*_m_ and ^app^*V*_max_ values of 3.6 μM and 7.6 nM/min, respectively (Fig. 1I).

The report that ARN726 marginally effects AC activity (44) prompted us to test it on AC in our experimental conditions using A375 cell lysates. As shown in Fig. 1J, more than 95% of hydrolysis of RBM1-151 was blocked by SOCLAC in both WT and AC-overexpressing A375 cells, indicating very low NAAA activity in our experimental conditions. The result that ARN726 provoked a significant reduction in RBM1-151 hydrolysis supports that, in agreement with the literature (44), ARN726 also inhibits AC. In the light of these results, ARN726 was disregarded as a tool to estimate AC, NAAA, and FAAH activities in intact cells (see below). The fact that RBM1-151 is hydrolyzed by AC, NAAA, and FAAH raised the possibility of estimating the three activities (AC, NAAA, and FAAH) in intact cells by using RBM1-151 in the presence of specific inhibitors, avoiding the need to utilize different substrates for each enzyme.

### RBM1-151 as a probe to monitor AC, FAAH, and NAAA in intact cells

**Fig. 2:**
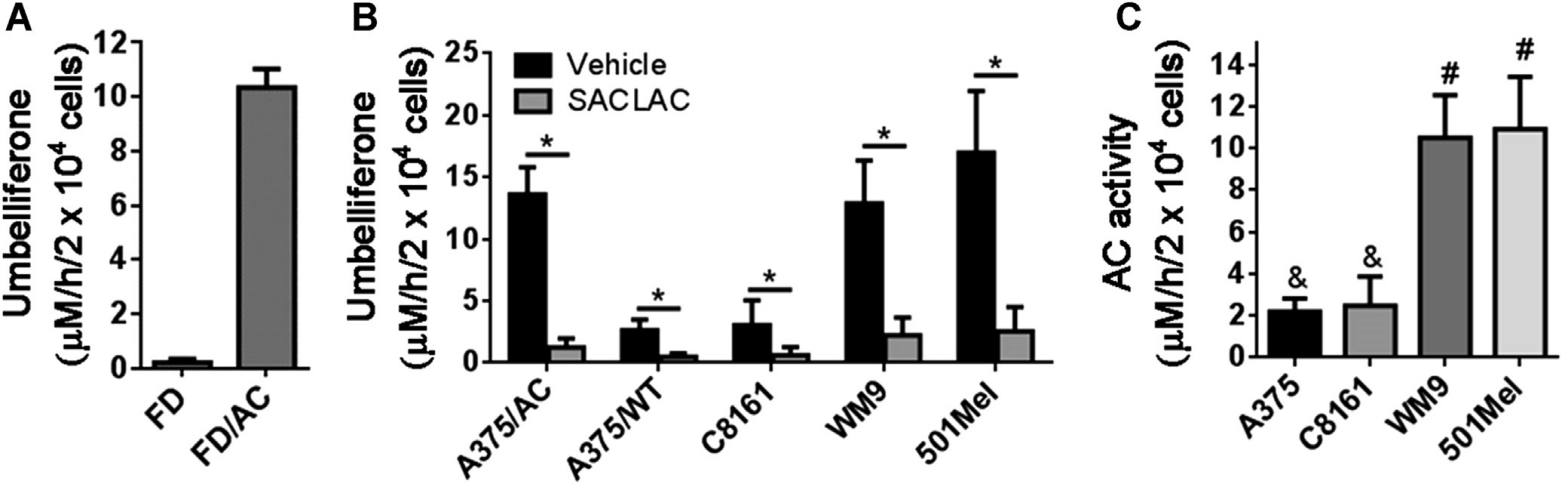
Hydrolysis of RBM1-151 in intact Farber (A) and melanoma (B, C) cells. The indicated cells were incubated with RBM1-151 (20 μM) for 3 h and further processed as indicated in the Materials and methods section. Data (mean ± SD) are from three experiments with triplicates. B: Asterisk indicates statistically significant difference at *P* < 0.0001 (unpaired, two tailed *t*-test between vehicle and SACLAC for each cell line). C: Different symbols denote statistically significant difference at *P* < 0.0001 by one-way ANOVA followed by Tukey multiple comparison test. A375/AC, AC-overexpressing A375 cells; A375/WT, A375 cells with AC basal levels; FD, Farber cells; FD/AC, Farber cells transduce to overexpress AC; SACLAC, (2*S*,3*R*)2-chloro-*N*-(1,3-dihydroxyoctadecan-2-yl)acetamide.

RBM1-151 was next tested in intact cells. As shown in Fig. 2A, minimal fluorescence was produced from RBM1-151 in Farber cells lacking AC, whereas the fluorophore was released from the same cells transduced for AC overexpression (FD/AC). In addition, higher fluorescence was produced in A375/AC than in A375/WT cells, and in both cases, fluorescence was significantly decreased to near background levels upon SOCLAC preincubation (Fig. 2B). RBM1-151 was also tested in other melanoma cell lines: C8161, WM9, and 501Mel. In these cell lines, SOCLAC pretreatment resulted in an 80–84% reduction in RBM1-151 hydrolysis. Together with A375, C8161 have an invasive phenotype, whereas WM9 and 501Mel show a proliferative phenotype. As shown in Fig. 2C, the two proliferative cell models exhibited a significant 4-fold higher AC activity than the invasive models.

**Fig. 3:**
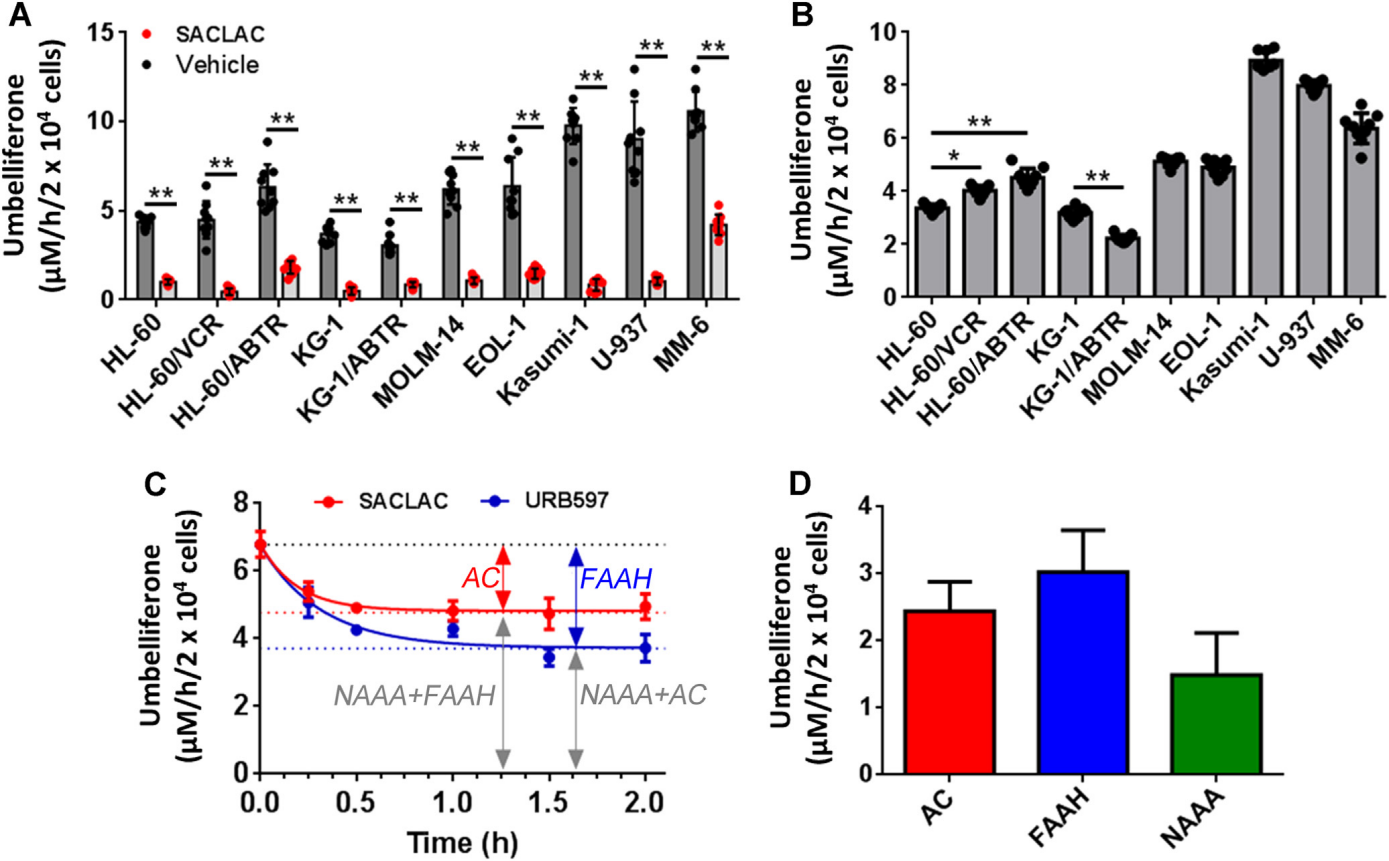
Amidase activity of different AML cell lines over RBM1-151. A: Cells were treated with SACLAC (15 μM) or vehicle (DMSO) for 1 h followed by 1 h incubation with RBM1-151 (20 μM). Cells were then processed for fluorescence analysis as detailed in the Materials and methods section. Data correspond to the mean ± SD of three experiments in triplicate. Asterisks indicate statistical difference at *P* ≤ 0.0001 (unpaired, two-tailed *t*-test vs. vehicle). B: AC activity. In the data of panel A, each SACLAC value was subtracted from the mean DMSO value. Asterisks indicate statistical difference at ∗*P* ≤ 0.001 and ∗∗*P* ≤ 0.0001 (one-way ANOVA, Tukey multiple comparisons test). C: MM-6 cells were treated with SACLAC (2.5 μM) or URB597 (50 μM) for the indicated times. The media were renewed, and hydrolysis of RBM1-151 (15 μM, 1 h) was determined as detailed in the Materials and methods section. The data (mean ± SD of one representative experiment with triplicates) were analyzed by the one-phase decay equation to afford the Y0 and plateau values. D: Estimated AC activity: 2.44 ± 0.44 μM/h/2 × 10^4^ cells (Y0—plateau in SACLAC treatment); estimated FAAH activity: 3.03 ± 0.62 μM/h/2 × 10^4^ cells (Y0—plateau in URB597 treatment) and estimated NAAA activity: 1.49 ± 0.63 μM/h/2 × 10^4^ cells (plateau in SACLAC treatment—FAAH activity) or 1.44 ± 0.65 μM/h/2 × 10^4^ cells (plateau in URB597 treatment—AC activity). AC, acid ceramidase; AML, acute myeloid leukemia; FAAH, fatty acid amide hydrolase; NAAA, *N*-acylethanolamine-hydrolyzing acid amidase; SACLAC, (2*S*,3*R*)2-chloro-*N*-(1,3-dihydroxyoctadecan-2-yl)acetamide.

AC activity was screened using RBM1-151 in a panel of human AML cell lines: MOLM-14, EOL-1, Kasumi-1, U-937, MM-6, HL-60 cells, HL-60 cells with acquired resistance to vincristine (HL-60/VCR) or ABT-737 (HL-60/ABTR), KG-1 cells, and KG-1 cells with acquired resistance to ABT-737 (KG-1/ABTR). Figure 3A shows the umbelliferone concentration in DMSO (vehicle) and SACLAC-treated cells, whereas Fig. 3B shows the difference between DMSO and SACLAC, which corresponds to AC activity (although 15 μM SACLAC was used in these screening experiments, latter concentration-response curves (supplemental Fig. S3) showed that 2.5 μM already induced a similar inhibition). In addition, AC protein content from these cells was analyzed by Western blot, and the data were significantly correlated with the amount of fluorophore that was inhibited by SACLAC treatment (supplemental Fig. S4) (Pearson correlation: *P* = 0.035; *R*^2^ = 0.44). The moderate correlation coefficient may be due to the fact that Western blot is semiquantitative and RBM1-151 uptake and trafficking to the lysosome may vary between cell lines. The highest AC activity (signal that is inhibited upon SACLAC treatment) was elicited by Kasumi-1, U-937, and MM-6 cells, followed by MOLM-14 and EOL-1 cells and finally, HL-60 and KG-1 cells.

We next assessed the utility of RBM1-151 in combination with specific inhibitors to monitor for AC, NAAA, and FAAH activities in intact cells. MM-6 cells, which retained a 40% of activity over RBM1-151 after SACLAC pretreatment, were used as model. To discern between activities, SACLAC and URB597 were used as inhibitors (ARN726 was disregarded given its inhibitory activity on both NAAA and AC). First, MM-6 cells were exposed to different concentrations of SACLAC. As shown in supplemental Fig. S3 the hydrolysis of RBM1-151 decreased dose-dependently until reaching a plateau at ∼30% of vehicle control cells with a 2.5 μM SACLAC dose. In the case of URB597, RBM1-151 processing also underwent a dose-dependent decline, but the plateau was not reached. Higher URB597 concentrations (>100 μM) could not be used as they compromised cell viability. As irreversible inhibitors, both SACLAC and URB597 induced a time-dependent inhibition of RBM1-151 hydrolysis (Fig. 3C). Data analysis by the one-phase decay equation allowed us to estimate AC activity (Y0—plateau in SACLAC treatment; 2.44 ± 0.44 μM/h/2 × 10^4^ cells), FAAH activity (Y0—plateau in URB597 treatment; 3.03 ± 0.62 μM/h/2 × 10^4^ cells), and NAAA activity (plateau in SACLAC treatment—FAAH activity; 1.49 ± 0.63 μM/h/2 × 10^4^ cells or plateau in URB597 treatment—AC activity; 1.44 ± 0.65 μM/h/2 × 10^4^ cells) (Fig. 3D). These overall results support the use of RBM1-151 in combination with AC and FAAH irreversible inhibitors to assess the activity of AC, NAAA, and FAAH in intact cells. The main advantage of this procedure is that changes in the three enzyme activities in response to large sets of treatments and conditions can be investigated in high-throughput formats using a single substrate. Unfortunately, this probe cannot be utilized in live-cell imaging. Both RBM1-151 and its amidase product (RBM1-151-NH2) are weakly fluorescent and, although umbelliferone, generated by RBM1-151 hydrolysis and further in situ oxidation, is strongly fluorescent, it is not retained in cells but diffuses to the media. A similar probe useful for live-cell imaging was reported (26).

## Discussion

Given the importance of AC in both inherited diseases and cancer progression, the availability of methods for the easy and specific detection of AC in intact cells is of high translational relevance. Several AC assays have been reported (1), including the fluorogenic methods developed in our laboratories based on the use of coumarin analogs of ceramides that release umbelliferone upon enzyme hydrolysis and mild oxidation (34). Among these, RBM14-C12 has been extensively used to measure AC activity in both cell-free systems and live cells (45, 46, 47). However, RBM14-C12 is also deamidated by NC, ACER3, and NAAA (35). Although high AC selectivity is achieved in cell lysates by using the appropriate buffer and pH, complete selectivity is challenging in live cells. In a previous article, we reported that C1-deoxydihydroceramides were specific AC substrates, although their metabolism by other amidases like FAAH and NAAA was not investigated (26). RBM1-151 is a new generation of fluorogenic AC substrates arisen from our previous research on this field. Amongst the ceramidase family of enzymes, RBM1-151 is only hydrolyzed by AC with a ^app^*K*_m_ 7.0 μM, lower than that reported for RBM14-C12 (25.9 μM) (34). However, FAAH and NAAA also accept RBM1-151 as a substrate. Although the *K*_m_ values for AC and FAAH are similar, the affinity of RBM1-151 for NAAA is 100 and 200 times higher than for AC and FAAH, respectively. Since cell lysates were used to determine the kinetic constants, conclusions on the maximum velocity cannot be drawn as they depend on the enzyme content of the cells. Importantly, we show here that each enzyme activity can be discerned by using specific irreversible AC and FAAH inhibitors.

AC is upregulated in AML (11) and promotes drug resistance (48), highlighting its potential as a therapeutic target in this leukemia (13, 49). We previously demonstrated that AC protein expression was 1.6- and 1.9-fold higher in drug-resistant HL-60/VCR and HL-60/ABTR cells, respectively, as compared with parental HL-60 cells, which correlated with higher P-gp/MDR-1 protein levels, increased drug efflux, and drug resistance (48). In agreement, in this work, we found that protein levels and SACLAC-sensitive activity (corresponding to AC) of those three cell lines over RBM1-151 were significantly higher in HL-60/VCR and HL-60/ABTR than in HL-60 cells. Intriguingly, in this study, AC activity (Fig. 3B) and AC protein levels (supplemental Fig. S2A, B) were lower in KG-1/ABTR than in KG-1 cells, which highlights the complexity of AML and the diverse response of different cell types to continuous drug-selection pressure.

Although the role of AC in the progression and response to treatment of AML cells has been reported, whether FAAH and NAAA are important in AML development and therapy has not been studied. Indeed, some AML cells retained RBM1-151 hydrolytic activity after SACLAC pretreatment, with SACLAC-resistant RBM1-151 hydrolysis being the highest in MM-6 cells. The use of URB597 together with SACLAC allowed the estimation of AC, FAAH, and NAAA activities in this cell line with RBM1-151. Collectively, these data are a proof of concept that combining RBM1-151 with irreversible AC and FAAH enzyme inhibitors allows the estimation of AC, FAAH, and NAAA activities in intact cells.

Similarly, AC is overexpressed in melanoma (9) and involved in melanoma drug sensitivity (10, 50), invasiveness (7, 8), and the ability to form cancer-initiating cells (8). Using RBM1-151 in the presence and absence of SACLAC, we have found that, amongst the cell lines investigated, the proliferative cell models exhibited a higher AC activity than the invasive models. High AC activity drives the ceramide-S1P rheostat toward S1P, a proliferative lipid mediator that counteracts the activities of ceramides as inducers of programmed cell death (51). Although the cannabinoid system has been reported to play a role in melanoma (52), only two articles have addressed the importance of FAAH and NAAA in melanoma progression. Hamtiaux *et al.* (53) reported that the treatment with PEA and FAAH inhibitors, including URB597, increased PEA levels and considerably reduced cell viability in B16 murine melanoma cells (53). The cell death increase observed with this combination of molecules was confirmed in vivo where only cotreatment with both PEA and URB597 decreased melanoma progression. In contrast, inhibition of NAAA with *N*-cyclohexanecarbonylpentadecylamine had no potentiating effect on cytotoxicity when added to PEA. This study suggested the interest of targeting FAAH in the management of melanoma and underlines the advantage of associating endocannabinoids with enzymatic hydrolysis inhibitors (53). On the other hand, Adinolfi *et al.* (52) showed that A375 cells express FAAH, that the FAAH substrate anandamide induces cytotoxicity against human melanoma cells in the micromolar range of concentrations, and that this cytotoxicity is potentiated by URB597 (52). Although the role of AC in melanoma progression is well sustained, more studies are necessary to unveil the putative importance of FAAH and NAAA in this disease. The use of RBM1-151 may be of help in these investigations.

In conclusion, by combining the fluorogenic amidase substrate RBM1-151 with specific inhibitors, we have developed a system to monitor AC, FAAH, and NAAA in intact cells. The system can be useful both in basic biological studies and in diagnostic and/or prognostic evaluations. Confirmation of the latter awaits determinations in patient samples.

## Data availability

All data generated or analyzed during this study are included in the article and Supplemental data.

## Supplemental data

This article contains supplemental data (29, 32, 34, 35, 40).

## Conflict of interest

The authors declare that they have no conflicts of interest with the contents of this article.
