# Supplementary material for: A fluorogenic substrate for the detection of lipid amidases in intact cells
Source: J Lipid Res. 2024 Feb 17;65(3):100520. doi: 10.1016/j.jlr.2024.100520 (PMC10956054; doi:10.1016/j.jlr.2024.100520)
Supplement: Supplemental data [file mmc1.docx]

**A fluorogenic substrate for the detection of lipid amidases in intact cells**

Mireia Casasampere,^1,#^ Johnson Ung,^2,3,#^ Alejandro Iñáñez,^1^ Carine Duffau,^4,5^ Kazuhito Tsuboi,^6^ Josefina Casas,^1,7^ Su-Fern Tan,^2,8^ David J. Feith,^2,8^ Nathalie Andrieu-Abadie,^4,5^ Bruno Segui,^4,5,9^ Thomas P. Loughran Jr,^2,8^ José Luis Abad^1,*^ and Gemma Fabrias^1,7,10,*^

^#^Equal first author

^*^To whom correspondence should be addressed

**Supplementary Methods**

**Synthesis of RBM1-151**

***tert*-Butyl (*S*)-[1-(methoxy(methyl)amino)-1-oxopropan-2-yl]carbamate (2)**

To a stirred solution of *N*-Boc L-alanine (10.00 g, 52.85 mmol) in anhydrous CH_2_Cl_2_ (300 ml) containing *N*,*O*-dimethylhydroxylamine hydrochloride (6.19 g, 63.42 mmol) and NMM (7 mL, 63.42 mmol) was portionwise added EDC·HCl (12.16 g, 63.42 mmol) at –15 °C. Then, the reaction mixture was slowly allowed to warm to rt while stirring over the course of 4 h. The reaction mixture was poured onto ice-cooled 1.0 M aq. HCl solution (100 mL), followed by extraction with CH_2_Cl_2_ (3 x 80 mL). The combined organic layers were washed with brine (3 x 70 mL), dried over MgSO_4_, filtered, and concentrated *in vacuo* to give the crude Weinreb amide (**2**) as a white solid (11.85 g, 97 %).

[α]_20_^D^ = -28.0 (c=1, MeOH). Lit.* [α]_20_^D^ = -28.0 (c= 1.0, MeOH).

^1^H NMR (400 MHz, CDCl_3_) *δ* 5.30-5.20 (m, 1H), 4.72-4.63 (m, 1H), 3.76 (s, 3H), 3.20 (s, 3H), 1.43 (s, 9H), 1.31 (d, *J* = 7.0 Hz, 3H).

***tert*-Butyl [(2*S*,3*R*)-3-hydroxyhex-5-en-2-yl]carbamate (4)**

Allylmagnesium chloride (6.9 mL, 13.8 mmol, 2.0 M in THF) was added dropwise to a solution of **2** (2.50 g, 6.90 mmol) in THF (30 mL) at -20 ºC. The reaction mixture was allowed to warm to 0 ^o^C over 2 h with stirring and was then quenched by addition to a 50 mL aqueous solution generated by 35 mL of water, 3.15 gr of citric acid (15 mmol) and 15 mL of 1N HCl aqueous solution. The resulting mixture was extracted with EtOAc (3 x 50 mL) and the combined organic layers were washed with saturated NaHCO_3_ (2 x 20 mL) aqueous solution and water (2 x 20 mL), dried over anhydrous MgSO_4_, filtered, and concentrated under reduced pressure. The crude residue was dissolved in ethanol (15 mL) and was added dropwise to a suspension of lithium tri-*tert*-butoxyaluminum hydride (3.86 g, 15.18 mmol) in ethanol (60 mL) at -78 ^o^C. After stirring at the same temperature for 30 min, the reaction mixture was allowed to warm to 0 ºC and was quenched with 10 % (w/v) aqueous citric acid (10%, 40 mL). The resulting mixture was carefully concentrated under reduced pressure and then extracted with EtOAc (3 x 50 mL). The organic layers were combined, washed with brine (2 x 50 mL), dried over anhydrous MgSO_4_, filtered, and concentrated in vacuo to give a 96:4 *anti*/*syn* crude mixture of diastereomers. Flash chromatography of the residue (gradient from 0 to 9 % of EtOAc in hexane) gave pure **4** (1.85 g, 78 %) as a colourless oil.

[α]_20_ ^D^ = +32.6 (c 1.0, CHCl_3_) [lit. (1) [α]_20_^D^ = +32.2 (c 1.32, CHCl_3_)].

^1^H NMR (400 MHz, CDCl_3_) *δ* 5.84 (ddt, *J* = 14.2, 10.2, 7.0 Hz, 1H), 5.16 (br d, *J* = 9.6 Hz, 1H), 5.12 (br s, 1H), 4.77 (s, 1H), 3.82-3.62 (m, 2H), 2.35-2.10 (m, 2H), 1.44 (s, 9H), 1.12 (d, 6.8 Hz, 3H).

^13^C NMR (101 MHz, CDCl_3_) *δ* 155.9, 134.8, 118.1, 79.6, 73.4, 50.3, 38.4, 28.5, 26.0, 14.6.

HRMS calcd. for C_17_H_35_NO_4_NaSi ([M ^+^ Na]^+^): 368.2233, found: 368.2236

**7‒(Allyloxy)‒2*H*‒chromen‒2‒one (6)**

To a stirred mixture of umbelliferone (3.0 g, 18.5 mmol) and K_2_CO_3_ (3.58 g, 25.9 mmol) in acetone (75 mL) was added dropwise allyl bromide (3.36 mL, 38.9 mmol). The reaction mixture was stirred under reflux for 4 h and the reaction evolution was monitored by TLC (hexanes/EtOAc 7:3). Then the reaction mixture was cooled to rt, diluted with water (50 mL) and extracted with EtOAc (3 x 40 mL). The combined organic layers were washed with brine (2 x 30 mL), dried over anhydrous MgSO_4_, filtered and concentrated under reduced pressure. Recrystallization of the residue from methanol in two consecutive crops yielded the wanted allyl ether derivative (3.29 g, 88 %) as creamy‒yellow crystals.

^1^H NMR (400 MHz, CDCl_3_) *δ* 7.63 (d, *J* = 9.5 Hz, 1H), 7.37 (d, *J* = 8.6 Hz, 1H), 6.86 (dd, *J* = 8.4, 2.4 Hz, 1H), 6.81 (d, *J* = 2.4 Hz, 1H), 6.25 (d, *J* = 9.5 Hz, 1H), 6.04 (ddt, *J* = 17.2, 10.6, 5.3 Hz, 1H), 5.44 (ddd, *J* = 17.3, 3.0, 1.5 Hz, 1H), 5.37 (dq, *J* = 10.5, 1.3 Hz, 1H), 4.60 (dt, *J* = 5.3, 1.0 Hz, 2H).

^13^C NMR (101 MHz, CDCl_3_) *δ* 161.7, 161.1, 155.7, 143.4, 132.1, 128.8, 118.4, 113.03,

112.96, 112.6, 101.7, 69.2.

HRMS calcd. for C_12_H_11_O_3_^+^ ([M ^+^ H]^+^): 203.0708, found: 203.0701.

***tert*-Butyl {(2*S*,3*R*,*E*)-3-hydroxy-7-[(2-oxo-2H-chromen-7-yl)oxy]hept-5-en-2-yl} carbamate (7)**

To a stirred solution of **4** (1,7 gr, 7.87 mmol) and **6** (4.77 gr, 23.5 mmol) in degassed CH_2_Cl_2_ (80 mL), 140 mg of 2^nd^ generation Grubbs catalyst was added portionwise at rt. The resulting mixture was stirred under refluxed in the darkness for 4 h, cooled down to rt and concentrated *in vacuo* to afford a crude, which was purified by flash chromatography on silica gel using two stepwise gradients (from 0 to 60 % EtOAc in hexanes, 1% each and from 0 to 2 % MeOH in CH_2_Cl_2_, 0.1% each) to afford pure **7** (1.69 g, 55% yield)

[α]_20_^D^ = -10.00 (c=1, CHCl_3_).

^1^H NMR (400 MHz, CDCl_3_) *δ* 7.63 (d, *J* = 8.8 Hz, 1H), 7.37 (d, *J* = 8.4 Hz, 1H), ), 6.85 (dd, *J* = 8.4, 2.4 Hz, 1H), 6.81 (d, *J* = 2.4 Hz, 1H), 6.25 (d, *J* = 9.6 Hz, 1H), 5.94 (dt, *J* = 16.0, 7.2 Hz, 1H), 5.81 (dt, *J* = 15.6, 5.6 Hz, 1H), 4.72 (br s, 1H), 4.57 (d, *J* = 5.6 Hz, 2H), 3.73 (m, 2H), 2.25 (m, 2H), 1.45 (s, 9H), 1.12 (d, 6.8 Hz, 3H).

^13^C NMR (101 MHz, CDCl_3_) *δ* 161.9, 161.4, 155.8, 143.6, 132.2, 128.9, 126.8, 113.09, 113.03, 112.6, 101.7, 79.6, 73.6, 69.0, 50.5, 28.4, 14.7.

HRMS calcd. for C_21_H_28_NO_6_^+^ ([M ^+^ H]^+^): 390.1911, found: 390.1907.

**7-{[(5*R*,6*S*,*E*)-6-amino-5-hydroxyhept-2-en-1-yl]oxy}-2H-chromen-2-one (8)**

A solution of **7** (1.22 g, 3.14 mmol) in MeOH (40 mL) was treated with neat acetyl chloride (1.2 mL) and the resulting mixture was stirred overnight in the darkness. The solvent was concentrated at reduced pressure and the residue was treated with 2 mL of a mixture of MeOH/30% NH_4_OH (1:1), evaporated *in vacuo (*twice) and the residue was purified by flash chromatography on silica gel using a stepwise gradient (from 0 to 20 % MeOH containing a 2% of aqueous 30% NH_4_OH) in CH_2_Cl_2_) to afford **8** (0.80 g, 88 %) as a white wax.

[α]_20_^D^ = +3.0 (c=1, CHCl_3_).

^1^H NMR (400 MHz, CD_3_OD) *δ* 7.88 (d, *J* = 9.6 Hz, 1H), 7.53 (d, *J* = 8.8 Hz, 1H), ), 6.93 (dd, *J* = 8.4, 2.4 Hz, 1H), 6.90 (d, *J* = 2.4 Hz, 1H), 6.25 (d, *J* = 9.6 Hz, 1H), 5.90 (m, 2H), 4.64 (d, *J* = 5.2 Hz, 2H), 3.83 (m, 1H), 3.31 (m, 1H), 2.31 (m, 2H), 1.24 (d, 6.8 Hz, 3H).

^13^C NMR (101 MHz, CD_3_OD) *δ* 163.4, 163.3, 156.9, 145.8, 131.5, 130.5, 128.8, 114.3, 114.0, 113.3, 102.5, 71.1, 69.9, 52.1, 37.3, 11.9.HRMS calcd. for C_16_H_20_NO_4_^+^ ([M ^+^ H]^+^): 290.1387, found: 290.1391.

***N*-{(2*S*,3*R*,*E*)-3-hydroxy-7-[(2-oxo-2H-chromen-7-yl)oxy]hept-5-en-2 yl}dodecanamide (RBM1-151)**

Lauric acid (71 mg, 0.35 mmol), HOBt (57 mg, 0.42 mmol, 1.2 equiv) and EDC·HCl (97 mg, 0.49 mmol, 1.4 equiv) were sequentially mixed in anhydrous CH_2_Cl_2_ (3 mL), and the resulting mixture was vigorously stirred at rt under argon atmosphere. After 5 min, the previous mixture was added dropwise to a solution of **8** (88 mg, 0.30 mmol) and TEA (100 μL, 2 equiv) in anhydrous CH_2_Cl_2_ (4 mL), and the reaction was stirred at rt for 12 h. The mixture was next diluted with CH_2_Cl_2_ (5 mL) and washed with brine (2 x 5 mL). The organic layer was dried over MgSO_4_, filtered, and the volatiles were removed under reduced pressure. Purification of the crude material by flash column chromatography on silica gel using a stepwise gradient (0.1% from 0 to 3 % MeOH in CH_2_Cl_2_) afforded 98 mg (70% yield) of **RBM1-151**.

[α]_20_^D^ = -23.0 (c=1, CHCl_3_).

^1^H NMR (400 MHz, CDCl_3_) *δ* 7.63 (d, *J* = 9.2 Hz, 1H), 7.37 (d, *J* = 8.4 Hz, 1H), ), 6.84 (dd, *J* = 8.4, 2.4 Hz, 1H), 6.80 (d, *J* = 2.4 Hz, 1H), 6.25 (d, *J* = 9.6 Hz, 1H), 5.93 (dt, *J* = 16.0, 7.2 Hz, 1H), 5.81 (dt, *J* = 15.6, 5.6 Hz, 1H), 5.72 (br s, 1H), 4.56 (d, *J* = 5.6 Hz, 2H), 4.04 (dquin, *J* = 7.6, 2.4 Hz, 2H), 3.74 (m, 2H), 2.87 (m, 1H), 2.25 (m, 2H), 2.18 (t, *J* = 7.6 Hz, 2H), 1.62 (m, 2H), 1.39-1.18 (br s, 16H), 1.14 (d, 6.8 Hz, 3H), 0.87 (t, 6.8 Hz, 3H).

^13^C NMR (101 MHz, CDCl_3_) *δ* 173.6, 161.9, 161.4, 155.8, 143.6, 132.2, 128.9, 126.8, 113.1, 113.0, 112.6, 101.7, 73.6, 69.0, 49.6, 36.9, 36.7, 32.0, 29.67, 29.66, 29.6, 29.43, 29.38, 29.36, 25.9, 22.7, 14.4, 14.2.

HRMS calcd. for C_28_H_42_NO_5_^+^ ([M ^+^ H]^+^): 472.3057, found: 472.3055.

**Supplementary Tables**

**Table S1.** Enzyme sources and buffers used in the study of RBM1-151 hydrolysis by AC, NC, ACER, NAAA and FAAH in cell free systems as presented in Figure 1.

| **Enzyme** | **Enzyme source** | **Buffer** | **Ref.** |
| --- | --- | --- | --- |
| AC | A375/AC cells, lysates | Acid buffer  (25 mM AcOH-NaAc, pH 4.6) | 1 |
| NAAA | HEK293/NAAA cells, lysates | Acid buffer  (25 mM AcOH-NaAc, pH 4.6) | 2 |
| NC | recombinant human NC | Neutral buffer A  (50 mM HEPES, 150 mM NaCl, 1% sodium cholate, pH 7.4) | 3 |
| FAAH | LNCaP cells, lysates  *ASAH2-*null MEFs, lysates | Neutral buffer B  (50 mM HEPES, 1 mM EDTA, 0.1% BSA, pH 7.4) | 4 |
| ACER1 | HeLa TRex ACER1 cells, microsomes | Alkaline buffer  (50 mM HEPES, 1 mM CaCl_2_, pH 9.0) | 5, 6 |
| ACER2 | HeLa TRex ACER2 cells, microsomes | Alkaline buffer  (50 mM HEPES, 1 mM CaCl_2_, pH 9.0) | 5, 6 |
| ACER3 | *ASAH2-*null MEFs, lysates | Alkaline buffer  (50 mM HEPES, 1 mM CaCl_2_, pH 9.0) | 6 |

AC, acid ceramidase; NC, neutral ceramidase; ACER1, alkaline ceramidase 1; ACER2, alkaline ceramidase 2; ACER3, alkaline ceramidase 3; NAAA, , *N*-acylethanolamine-hydrolyzing acid amidase; FAAH, fatty acid amide hydrolase.

References

1. Bedia et al. [A simple fluorogenic method for determination of acid ceramidase activity and diagnosis of Farber disease.](https://pubmed.ncbi.nlm.nih.gov/20871013/) J Lipid Res. 2010, 51, 3542-3547
2. Tsuboi et al. [Molecular characterization of N-acylethanolamine-hydrolyzing acid amidase, a novel member of the choloylglycine hydrolase family with structural and functional similarity to acid ceramidase.](https://pubmed.ncbi.nlm.nih.gov/15655246/) J. Biol. Chem. 2005, 25, 11082-11092.
3. Casasampere et al. New fluorogenic probes for neutral and alkaline ceramidases. J. Lipid Res. 2019, 60, 1174–11181.
4. Endsley et al. Expression and Function of fatty acid amide hydrolase in prostate cancer. Int. J. Cancer. 2008, 123, 1318–1326.
5. Xu et al. Golgi alkaline ceramidase regulates cell proliferation and survival by controlling levels of sphingosine and S1P. *FASEB J.* 2006, 20, 1813–1825.
6. Casasampere et al. [Activity of neutral and alkaline ceramidases on fluorogenic N-acylated coumarin-containing aminodiols.](https://pubmed.ncbi.nlm.nih.gov/26286360/) J Lipid Res. 2015, 56, 2019-2028.

**Supplementary Figures**

**

**

**Figure S1. A. Effect of different concentrations of SOCLAC and SACLAC on acid ceramidase activity.** Lysates (20 µg) of A375/AC cells were incubated with the inhibitors for 1 h and then with RBM1-151 for 3 h at acid pH (Table S1). The reaction mixture was then processed for fluorescence release as detailed in the methods section. Data are from 2 experiments with triplicates.

**
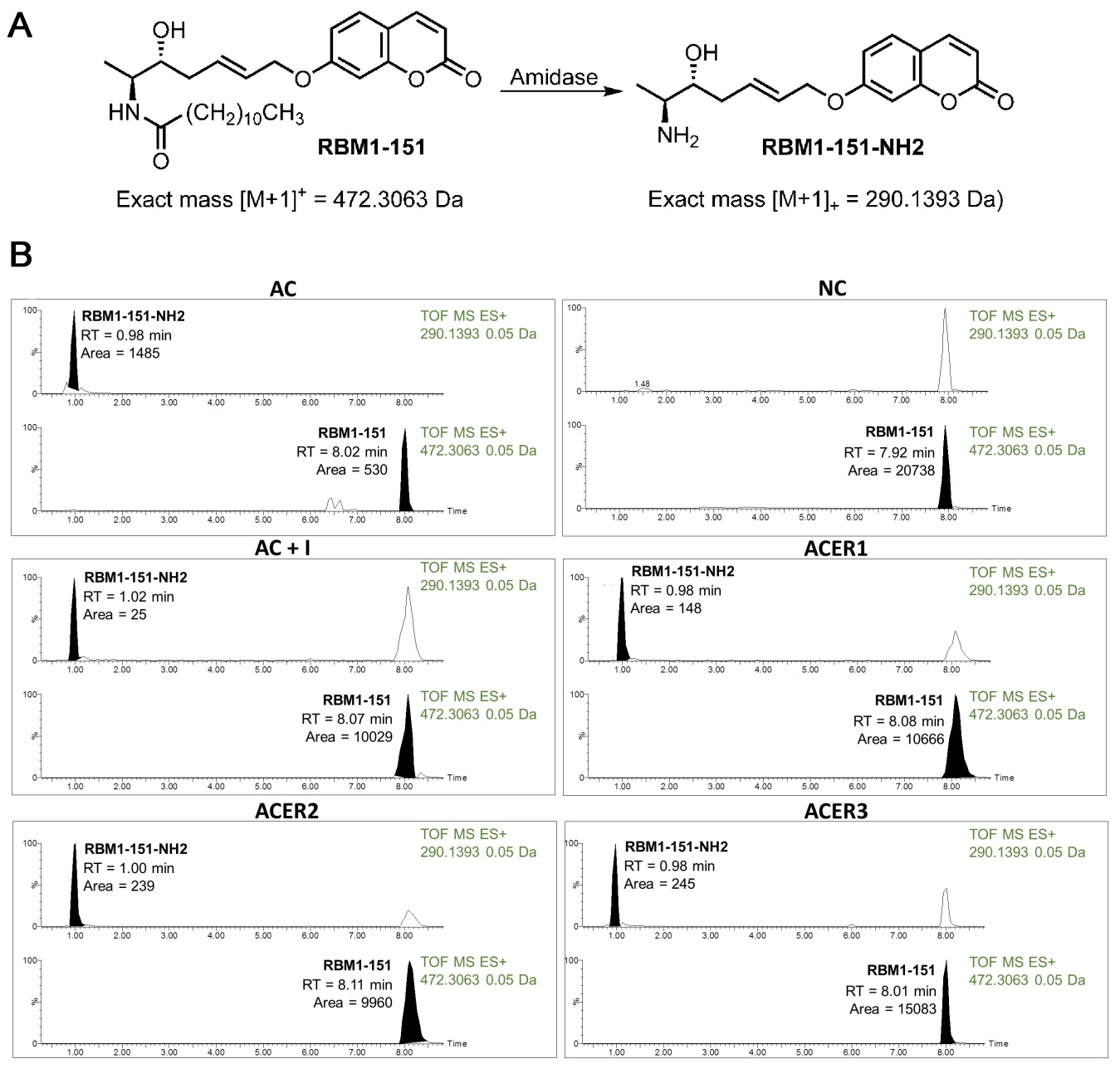
**

**Figure S1. Analysis of RBM1-151 hydrolysis by different ceramidases.** A. Reaction substrate and product. B. Representative UPLC-HRMS chromatograms. The retention times and areas corresponding to RBM1-151 (Exact mass [M+1]^+^ = 472.3063 ± 0.05 Da) and its hydrolysis product RBM1-151-NH2 (Exact mass [M+1]^+^ = 290.1393 ± 0.05 Da) are shown next to each peak. The means ± SD of all replicates are given in Figure 1A. AC, acid ceramidase; I, SACLAC (AC inhibitor); NC, neutral ceramidase; ACER1, alkaline ceramidase 1; ACER2, alkaline ceramidase 2; ACER3, alkaline ceramidase 3.


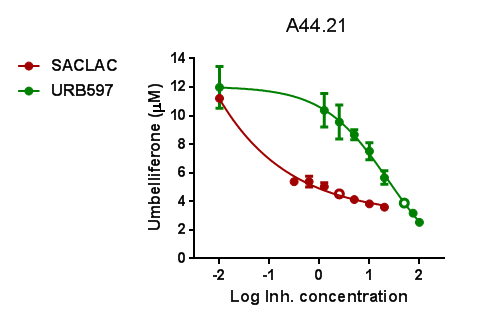


**Figure S3. Dose response of inhibition of RBM1-151 hydrolysis by SACLAC and URB597 in MM-6 cells.** Cells were treated with different concentrations of SACLAC (1/2 serial dilution from 20 to 0.3125 µM and 0.01 µM) or URB597 (3/4 serial dilution from 100 to 5 µM and 0.01 µM) for 1 h. The data correspond to mean ± SD of three experiments with triplicates. The data was analyzed using the “nonlinear-fit log(inhibitor) vs response – variable slope (four parameters)” function from Prism v10.0.2. The open circle in each curve corresponds to the concentration of inhibitor selected in the time course studies

**
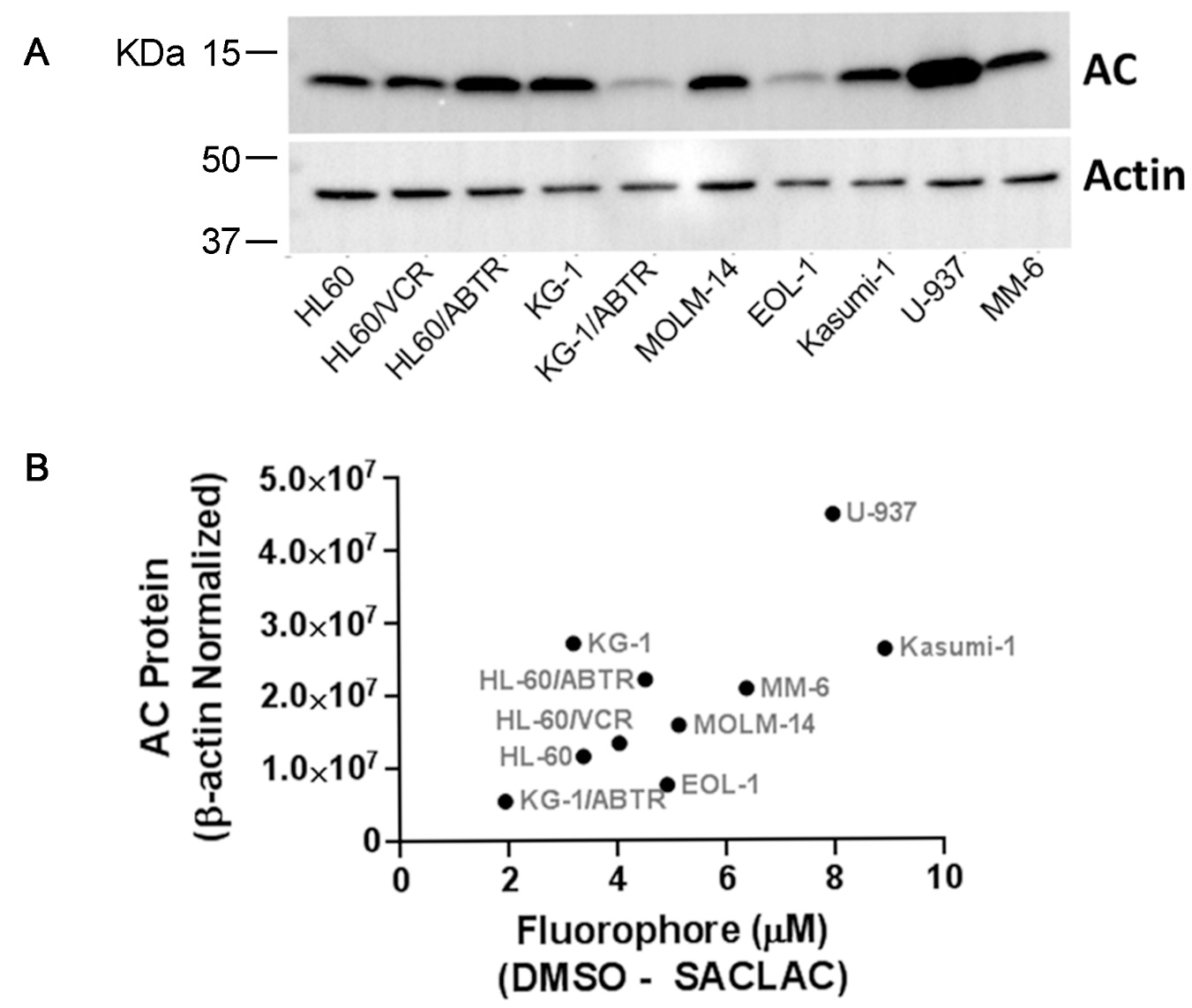
**

**Figure S4. Relationship between acid ceramidase immunoblotting and inhibition of RBM1-151 hydrolysis.** A. Representative western blot showing the acid ceramidase (AC) content in AML cell lines. B. Correlation between AC protein levels (normalized over β-actin) and AC activity (mean vehicle-mean SACLAC of Fig. 4, panel A). R^2^ = 0.44, *P* = 0.035 (Pearson correlation).

**NMR Spectra**

***tert*-Butyl [(2*S*,3*R*)-3-hydroxyhex-5-en-2-yl]carbamate (4)**

**^1^H NMR**

**COSY**

**^13^C NMR**

**DEPT 135**

**DEPT 135Q**

**DEPT 90**

**HSQC**

**7‒(Allyloxy)‒2*H*‒chromen‒2‒one (6)**

**^1^H NMR**

**COSY**

**^13^C NMR**

**DEPT 135**

**DEPT 135Q**

**DEPT 90**

**HSQC**

***tert-*Butyl {(2*S*,3*R*,*E*)-3-hydroxy-7-[(2-oxo-2H-chromen-7-yl)oxy]hept-5-en-2-yl} carbamate (7)**

**^1^H NMR**

**COSY**

**^13^C NMR**

**DEPT 135**

**DEPT 135Q**

**DEPT 90**

**HSQC**

**7-{[(5*R*,6*S*,*E*)-6-amino-5-hydroxyhept-2-en-1-yl]oxy}-2H-chromen-2-one (8)**

**^1^H NMR**

**COSY**

**^13^C NMR**

**DEPT 135**

**DEPT 135Q**

**DEPT 90**

**HSQC**

***N*-{(2*S*,3*R*,*E*)-3-hydroxy-7-[(2-oxo-2H-chromen-7-yl)oxy]hept-5-en-2 yl}dodecanamide (RBM1-151)**

**^1^H NMR**

**COSY**

**^13^C NMR**

**DEPT 135**

**DEPT 135Q**

**DEPT 90**

**HSQC**
